# Molecular characterization of H3N2 influenza A viruses isolated from Ontario swine in 2011 and 2012

**DOI:** 10.1186/s12985-014-0194-z

**Published:** 2014-11-22

**Authors:** Helena Grgić, Marcio Costa, Robert M Friendship, Susy Carman, Éva Nagy, Greg Wideman, Scott Weese, Zvonimir Poljak

**Affiliations:** Department of Population Medicine, Ontario Veterinary College, University of Guelph, Guelph, Ontario N1G 2 W1 Canada; Department of Pathobiology, Ontario Veterinary College, University of Guelph, Guelph, Ontario N1G 2 W1 Canada; Animal Health Laboratory, University of Guelph, Guelph, Ontario N1H 6R8 Canada; South-West Ontario Veterinary Services, Stratford, Ontario Canada; Centre for Public Health and Zoonoses, University of Guelph, Guelph, Canada

**Keywords:** Influenza A virus, Triple-reassortant H3N2, Swine, 454 sequencing, Phylogenetic analysis

## Abstract

**Background:**

Data about molecular diversity of commonly circulating type A influenza viruses in Ontario swine are scarce. Yet, this information is essential for surveillance of animal and public health, vaccine updates, and for understanding virus evolution and its large-scale spread.

**Methods:**

The study population consisted of 21 swine herds with clinical problems due to respiratory disease. Nasal swabs from individual pigs were collected and tested by virus isolation in MDCK cells and by rtRT-PCR. All eight segments of 10 H3N2 viruses were sequenced using high-throughput sequencing and molecularly characterized.

**Results:**

Within-herd prevalence ranged between 2 and 100%. Structurally, Ontario H3N2 viruses could be classified into three different groups. Group 1 was the most similar to the original trH3N2 virus from 2005. Group 2 was the most similar to the Ontario turkey H3N2 isolates with PB1 and NS genes originating from trH3N2 virus and M, PB2, PA and NP genes originating from the A(H1N1)pdm09 virus. All Group 3 internal genes were genetically related to A(H1N1)pdm09. Analysis of antigenic sites of HA1 showed that Group 1 had 8 aa changes within 4 antigenic sites, A(1), B(3), C(2) and E(2). The Group 2 viruses had 8 aa changes within 3 antigenic sites A(3), B(3) and C(2), while Group 3 viruses had 4 aa changes within 3 antigenic sites, B(1), D(1) and E(2), when compared to the cluster IV H3N2 virus [A/swine/Ontario/33853/2005/(H3N2)].

**Conclusions:**

The characterization of the Ontario H3N2 viruses clearly indicates reassortment of gene segments between the North American swine trH3N2 from cluster IV and the A(H1N1)pdm09 virus.

**Electronic supplementary material:**

The online version of this article (doi:10.1186/s12985-014-0194-z) contains supplementary material, which is available to authorized users.

## Background

Influenza viruses, belonging to the family *Orthomyxoviridae,* are enveloped viruses with segmented negative-sense RNA genome [1]. Influenza A viruses evolve rapidly, creating new variants which could be the result of either point mutations or reassortment. Eighteen hemagglutinin (HA) and 11 neuraminidase (NA) types have been reported to date, classifying viruses into subtypes H1 to H18 and N1 to N11 [2].

In 2005, the triple-reassortant H3N2 (trH3N2) virus was reported in Canada and spread widely, affecting swine industries in all provinces [3]. After initial detection of the trH3N2 virus in 2005, there were no further scientific publications about molecular diversity of influenza viruses circulating in Canadian swine until 2009 [4]. This information has been particularly limited in Ontario. According to a recent statistic from 2012 and 2013, Ontario is the province with the second largest number of pigs on farms in Canada, and with the largest number of farms with pigs [5]. This information is important for surveillance of influenza viruses and informing animal and public health decisions, vaccine updates, and for understanding virus evolution and its large-scale spread. Therefore, the objective of this study was to determine which H3N2 influenza A viruses circulated in Ontario swine in 2011 and 2012.

## Results

### Descriptive analysis

Most of the 21 herds included in this study were finisher sites only (n = 9), followed by nursery (n = 6), wean-to-finish (n = 3), farrow-to-finish (n = 1), farrow-to-grow (n = 1), and farrow-to-wean (n = 1) sites. Sow capacity ranged between 600 and 650 sows. Nursery inventory at the date of sampling ranged between 2000 and 2500 animals, and finisher pig inventory ranged between 950 and 5000 animals. The average number of samples tested per herd was 56 (7–100), and the average number of pooled real-time reverse transcription PCR (rtRT-PCR) tests was 10 (5–20) per herd. The mean number of sampled animals was 53 and 57.3 animals in influenza-virus-negative and influenza-virus-positive herds, respectively (p = 0.7).

In this study, 13 of 21 herds (61.9%) tested positive for influenza virus using virus isolation in Madin-Darby canine kidney (MDCK) cells, while 8 of 12 herds (66.7%) tested rtRT-PCR-positive on pooled samples. In total, 16 herds (76.2%) tested positive by either virus isolation or rtRT-PCR. Viruses from eleven out of 16 positive herds were typed as H3N2, from 3 herds were typed as H1N1, and in 2 herds, subtyping was not successful. In total 11 H3N2 isolates from 10 different herds were included for full genome sequencing in 2 separate runs. However, sequencing results for 10 isolates from 9 herds were obtained. Full genome sequencing results for one virus could not be obtained despite repeated inclusion of this virus in two separate runs.

**Figure 1:**
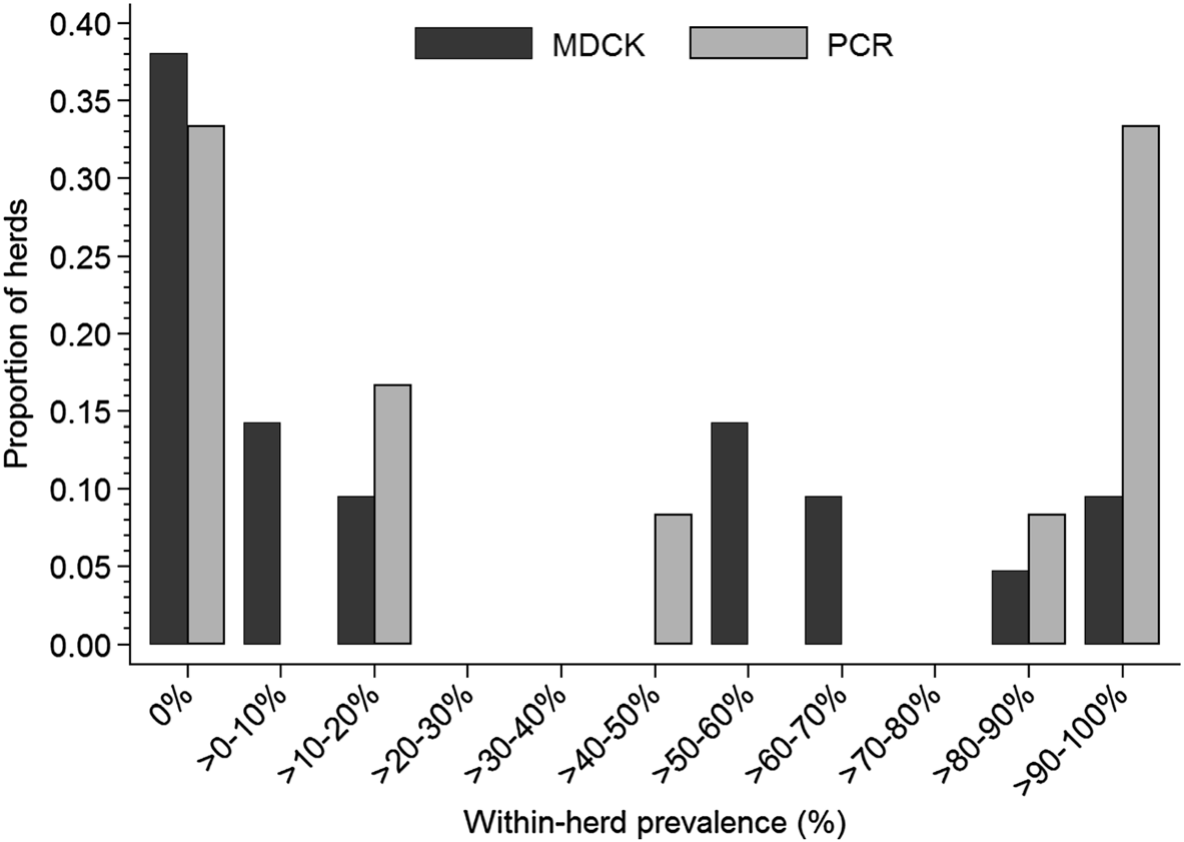
**Within-herd prevalence of positive samples for influenza virus during testing of individual samples by virus isolation in Madin-Darby canine kidney (MDCK) cells, and during testing of 3:1 pools of nasal swabs on real-time reverse transcription (rtRT)-PCR in Ontario swine herds during 2011–2012.**

Figure 1 depicts the within-herd prevalence of influenza virus shedding in 21 Ontario swine herds tested by MDCK and rtRT-PCR. The within-herd prevalence ranged between 2% and 100%. In herds that had at least one virus positive isolation, the mean within-herd prevalence was 84% in sow herds (n = 2), 46% in nursery herds (n = 6), 30% in finisher herds (n = 4), and 57% in wean-to-finish herds (n = 1).

### Genetic characterization of 10 Ontario H3N2 influenza viruses

**Figure 2:**
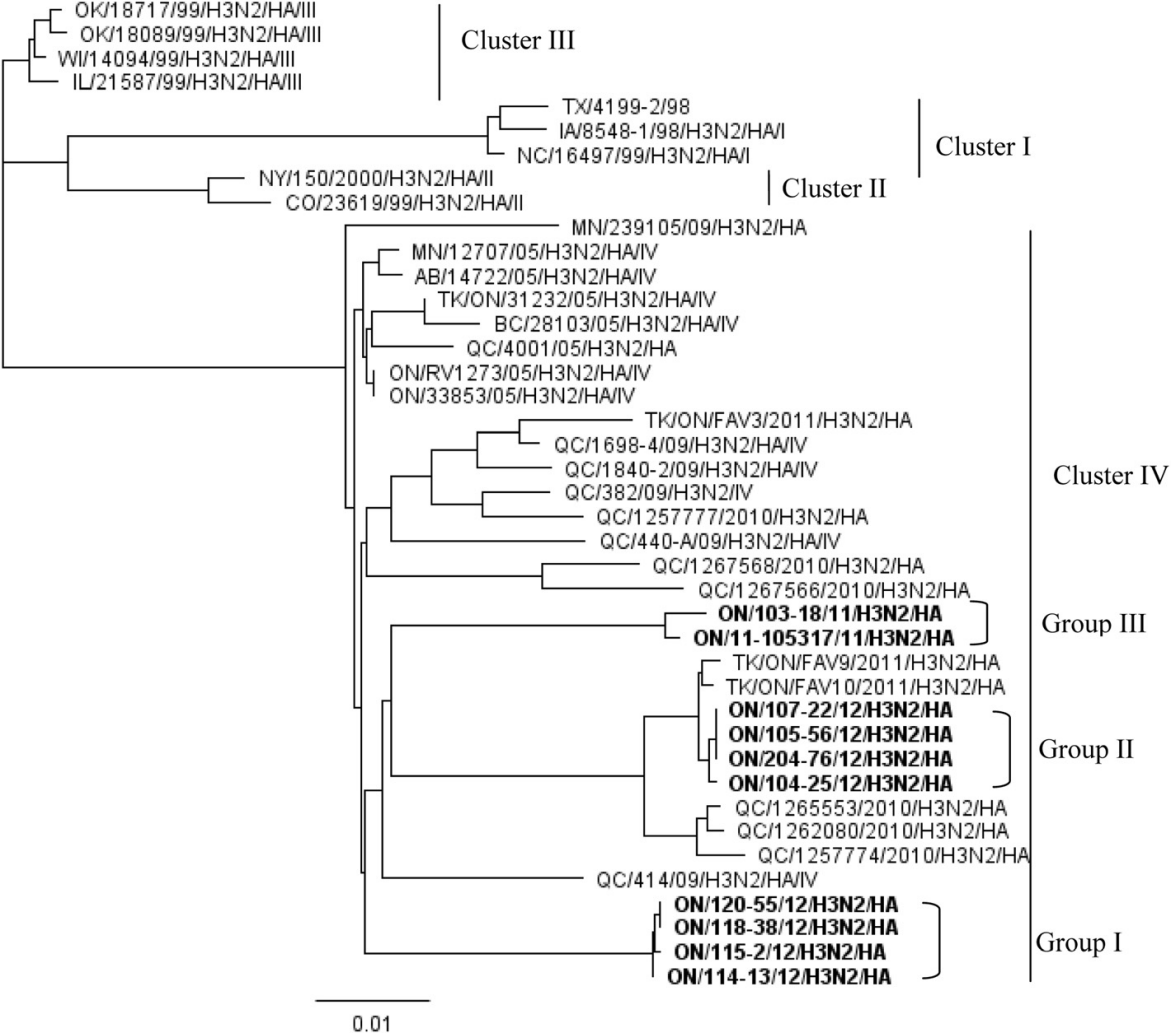
**Phylogenetic tree of the HA gene nucleotide sequences of 10 Ontario H3N2 viruses.**

Structurally, Ontario H3N2 viruses detected in this study could be classified into three groups. The first group consisted of 4 viruses in which all segments were similar to those of the trH3N2 virus that emerged in Ontario in 2005 [3]. The second group consisted of 4 viruses with HA, NA, PB1 and NS genes originating from the trH3N2 virus, and M, PB2, PA and NP genes originating from the pandemic A(H1N1)pdm09 virus. The third group consisted of 2 viruses with HA and NA genes originating from the trH3N2 virus, and with all internal genes originating from the A(H1N1)pdm09 virus. The phylogenetic relationships of the HA gene of these Ontario H3N2 viruses are presented, in association with other reference viruses in Figure 2. Genome constellations identified in Ontario H3N2 viruses isolated from swine in 2011 and 2012 are presented in Additional file 1. The GenBank accession numbers of the sequences reported in this paper are: [KF840476-KF840478, KJ413857-KJ413933].

More detailed analysis of HA and NA genes revealed that Group 1 indeed was the most similar to the original Cluster IV trH3N2 [A/swine/ON/33853/2005 (H3N2] virus from 2005, with 97.2% to 97.3% and 97.7% to 97.8% identity of HA and NA genes, respectively (Tables 1 and 2). Interestingly, when compared to published standards, the NA gene of these Group 1 viruses showed the highest identity (at 98.3% to 98.7%) (Table 2) with the two 2011 Ontario turkey isolates, while the HA gene of 4 Group 1 viruses showed much more modest identity with these 2 turkey viruses (94.6% to 94.7%) (Table 1). Group 1 was a homogenous group of 4 viruses, with 99.9% to 100% (HA) and 99.7% to 100% (NA) identity of the HA and NA genes, although these herds were not a part of the same production system.

**Table 1:** **Identity table of the full length of the HA gene of 10 Ontario H3N2 viruses isolated from swine in 2011–2012 and selected references viruses based on nucleotide sequence**

	**G1-A/sw/ON/114-13/12/H3N2/HA**	**G1-A/sw/ON/115-2/12/H3N2/HA**	**G1-A/sw/ON/118-38/12/H3N2/HA**	**G1-A/sw/ON/120-55/12/H3N2/HA**	**G2-A/sw/ON/104-25/12/H3N2/HA**	**G2-A/sw/ON/204-76/12/H3N2/HA**	**G2-A/sw/ON/105-56/12/H3N2/HA**	**G2-A/sw/ON/107-22/12/H3N2/HA**	**G3-A/sw/ON/103-18/11/H3N2/HA**	**G3-A/sw/ON/11-105317/11/H3/HA**
**G1-A/sw/ON/114-13/12/H3N2/HA**	100	99.9	99.9	99.9	94.8	94.8	94.8	94.8	94.9	95.2
**G1-A/sw/ON/115-2/12/H3N2/HA**	99.9	100	99.9	99.9	94.7	94.7	94.7	94.7	94.9	95.1
**G1-A/sw/ON/118-38/12/H3N2/HA**	99.9	99.9	100	100	94.7	94.7	94.7	94.7	94.9	95.1
**G1-A/sw/ON/120-55/12/H3N2/HA**	99.9	99.9	100	100	94.7	94.7	94.7	94.7	94.9	95.1
**G2-A/sw/ON/104-25/12/H3N2/HA**	94.8	94.7	94.7	94.7	100	99.9	99.9	99.9	94.9	94.9
**G2-A/sw/ON/204-76/12/H3N2/HA**	94.8	94.7	94.7	94.7	99.9	100	100	100	94.8	94.9
**G2-A/sw/ON/105-56/12/H3N2/HA**	94.8	94.7	94.7	94.7	99.9	100	100	100	94.8	94.9
**G2-A/sw/ON/107-22/12/H3N2/HA**	94.8	94.7	94.7	94.7	99.9	100	100	100	94.8	94.9
**G3-A/sw/ON/103-18/11/H3N2/HA**	94.9	94.9	94.9	94.9	94.9	94.8	94.8	94.8	100	99.5
**G3-A/sw/ON/11-105317/11/H3N2/HA**	95.2	95.1	95.1	95.1	94.9	94.9	94.9	94.9	99.5	100
**MN/12707/05/H3N2/HA/IV**	97	96.9	96.9	96.9	96.5	96.5	96.5	96.5	96.5	96.8
**AB/14722/05/H3N2/HA/IV**	97	96.9	96.9	96.9	96.5	96.5	96.5	96.5	96.5	96.8
**TK/ON/31232/05/H3N2/HA/IV**	96.9	96.8	96.8	96.8	96.4	96.4	96.4	96.4	96.4	96.6
**BC/28103/05/H3N2/HA/IV**	96.5	96.4	96.4	96.4	96	96	96	96	95.9	96.2
**QC/4001/05/H3N2/HA**	96.6	96.5	96.5	96.5	96.1	96.1	96.1	96.1	96.1	96.4
**ON/RV1273/05/H3N2/HA/IV**	97.3	97.2	97.2	97.2	96.8	96.8	96.8	96.8	96.8	97.1
**ON/33853/05/H3N2/HA/IV**	97.3	97.2	97.2	97.2	96.8	96.8	96.8	96.8	96.8	97.1
**QC/1698-4/09/H3N2/HA/IV**	96.1	96.1	96.1	96.1	95.5	95.5	95.5	95.5	95.6	95.9
**QC/1840-2/09/H3N2/HA/IV**	95.9	95.9	95.9	95.9	95.4	95.4	95.4	95.4	95.6	95.8
**QC/382/09/H3N2/IV**	96.1	96	96	96	95.5	95.5	95.5	95.5	95.5	95.7
**QC/1257777/2010/H3N2/HA**	95.8	95.8	95.8	95.8	95.2	95.2	95.2	95.2	95.2	95.4
**QC/440-A/09/H3N2/HA/IV**	95.6	95.6	95.6	95.6	95	95	95	95	95.2	95.4
**MN/239105/09/H3N2/HA**	95.8	95.8	95.8	95.8	95.3	95.3	95.3	95.3	95.5	95.8
**QC/414/09/H3N2/HA/IV**	95.7	95.6	95.6	95.6	95.4	95.4	95.4	95.4	95.7	95.9
**QC/1267568/2010/H3N2/HA**	95.2	95.1	95.1	95.1	94.6	94.6	94.6	94.6	94.8	95.1
**QC/1267566/2010/H3N2/HA**	95.1	95	95	95	94.3	94.3	94.3	94.3	94.3	94.5
**TK/ON/FAV9/2011/H3N2/HA**	94.7	94.7	94.7	94.7	99.7	99.7	99.7	99.7	94.8	94.9
**TK/ON/FAV10/2011/H3N2/HA**	94.7	94.6	94.6	94.6	99.6	99.6	99.6	99.6	94.8	94.9
**QC/1265553/2010/H3N2/HA**	94.6	94.6	94.6	94.6	98.6	98.6	98.6	98.6	94.8	94.9
**QC/1262080/2010/H3N2/HA**	94.5	94.5	94.5	94.5	98.8	98.8	98.8	98.8	94.6	94.8
**QC/1257774/2010/H3N2/HA**	94.6	94.5	94.5	94.5	98.7	98.7	98.7	98.7	94.4	94.6
**OK/18717/99/H3N2/HA/III**	94.2	94.2	94.1	94.1	93.7	93.8	93.8	93.8	93.7	93.9
**OK/18089/99/H3N2/HA/III**	94.2	94.1	94.1	94.1	93.6	93.8	93.8	93.8	93.7	94
**WI/14094/99/H3N2/HA/III**	94.2	94.2	94.2	94.2	93.9	94	94	94	93.7	94
**IL/21587/99/H3N2/HA/III**	94.1	94.1	94.1	94.1	93.8	93.9	93.9	93.9	93.6	93.9
**TX/4199-2/98**	90.4	90.3	90.3	90.3	90.1	90.3	90.3	90.3	89.4	89.7
**IA/8548-1/98/H3N2/HA/I**	90.5	90.4	90.4	90.4	90.2	90.4	90.4	90.4	89.4	89.7
**NC/16497/99/H3N2/HA/I**	90.7	90.7	90.7	90.7	90.5	90.7	90.7	90.7	90.1	90.3
**NY/150/2000/H3N2/HA/II**	93.1	93	93	93	92.4	92.5	92.5	92.5	92.4	92.5
**CO/23619/99/H3N2/HA/II**	92.7	92.7	92.7	92.7	92.1	92.2	92.2	92.2	92.1	92.3

**Table 2:** **Identity table of the full length of the NA gene of 10 Ontario H3N2 viruses isolated from swine in 2011–2012 and selected references viruses based on nucleotide sequence**

	**G1-A/sw/ON/114-13/12/H3N2/NA**	**G1-A/sw/ON/115-2/12/H3N2/NA**	**G1-A/sw/ON/118-38/12/H3N2/NA**	**G1-A/sw/ON/120-55/12/H3N2/NA**	**G2-A/sw/ON/104-25/12/H3N2/NA**	**G2-A/sw/ON/204-76/12/H3N2/NA**	**G2-A/sw/ON/105-56/12/H3N2/NA**	**G2-A/sw/ON/107-22/12/H3N2/NA**	**G3-A/sw/ON/103-18/11/H3N2/NA**	**G3-A/sw/ON/11-105317/11/H3/NA**
**G1-A/sw/ON/114-13/12/H3N2/NA**	100	99.7	99.9	99.9	98.5	98.6	98.6	98.6	96	96
**G1-A/sw/ON/115-2/12/H3N2/NA**	99.7	100	99.9	99.9	98.8	98.9	98.9	98.9	96.1	96.2
**G1-A/sw/ON/118-38/12/H3N2/NA**	99.9	99.9	100	100	98.7	98.7	98.7	98.7	96	96
**G1-A/sw/ON/120-55/12/H3N2/NA**	99.9	99.9	100	100	98.7	98.7	98.7	98.7	96	96
**G2-A/sw/ON/104-25/12/H3N2/NA**	98.5	98.8	98.7	98.7	100	99.9	99.9	99.9	96.3	96.4
**G2-A/sw/ON/204-76/12/H3N2/NA**	98.6	98.9	98.7	98.7	99.9	100	100	100	96.4	96.5
**G2-A/sw/ON/105-56/12/H3N2/NA**	98.6	98.9	98.7	98.7	99.9	100	100	100	96.4	96.5
**G2-A/sw/ON/107-22/12/H3N2/NA**	98.6	98.9	98.7	98.7	99.9	100	100	100	96.4	96.5
**G3-A/sw/ON/103-18/11/H3N2/NA**	96	96.1	96	96	96.3	96.4	96.4	96.4	100	99.9
**G3-A/sw/ON/11-105317/11/H3N2/NA**	96	96.2	96	96	96.4	96.5	96.5	96.5	99.9	100
**TK/ON/31232/05/H3N2/NA/IV**	97.5	97.7	97.5	97.5	98	98.1	98.1	98.1	98	98.1
**ON/33853/05/H3N2/NA/IV**	97.7	97.8	97.7	97.7	98.2	98.2	98.2	98.2	98	98.1
**QC/1698-4/09/H3N2/NA/IV**	96.2	96.3	96.2	96.2	96.7	96.7	96.7	96.7	96.9	97
**QC/382/09/H3N2/NA/IV**	96.5	96.7	96.5	96.5	97	97.1	97.1	97.1	97.3	97.4
**QC/1257777/2010/H3N2/NA**	96.2	96.4	96.2	96.2	96.9	97	97	97	96.9	97
**QC/1267566/2010/H3N2/NA**	95.6	95.7	95.6	95.6	96.1	96.2	96.2	96.2	97	97
**QC/1267568/2010/H3N2/NA**	95.3	95.5	95.3	95.3	95.8	95.9	95.9	95.9	96.2	96.2
**TK/ON/FAV9/2011/H3N2/NA**	98.3	98.6	98.4	98.4	99.4	99.4	99.4	99.4	96.1	96.2
**TK/ON/FAV10/2011/H3N2/NA**	98.4	98.7	98.6	98.6	99.5	99.6	99.6	99.6	96.3	96.3
**QC/1262080/2010/H3N2/NA**	95.5	95.6	95.5	95.5	96	96	96	96	95.5	95.6
**QC/1257774/2010/H3N2/NA**	95.3	95.5	95.3	95.3	95.8	95.9	95.9	95.9	95.7	95.7
**QC/1265553/2010/H3N2/NA**	95.4	95.5	95.4	95.4	95.9	96	96	96	95.5	95.5
**BC/28103/05/H3N2/NA/IV**	97.5	97.7	97.5	97.5	98	98.1	98.1	98.1	97.9	97.9
**MN/12707/05/H3N2/NA/IV**	96.9	97	96.9	96.9	97.4	97.4	97.4	97.4	97.2	97.3
**AB/14722/05/H3N2/NA/IV**	96.7	96.8	96.7	96.7	97.2	97.2	97.2	97.2	97	97.1
**OK/18717/99/H3N2/NA/III**	93.8	93.9	93.8	93.8	94.1	94.2	94.2	94.2	94.2	94.3
**IL/21587/99/H3N2/NA/III**	93.7	93.8	93.7	93.7	94	94.1	94.1	94.1	94.1	94.2
**WI/14094/99/H3N2/NA/III**	93.7	93.9	93.7	93.7	94.1	94.1	94.1	94.1	94.1	94.2
**OK/18089/99/H3N2/NA/III**	93.8	93.9	93.8	93.8	94.1	94.2	94.2	94.2	94.2	94.3
**NC/16497/99/H3N2/NA/I**	93.5	93.8	93.7	93.7	94	94.1	94.1	94.1	93.9	94
**TX/4199-2/98**	93.7	93.9	93.7	93.7	94.1	94.2	94.2	94.2	94.2	94.2
**IA/8548-1/98/H3N2/NA/I**	93.4	93.6	93.4	93.4	93.8	93.9	93.9	93.9	93.9	94
**CO/23619/99/H3N2/NA/II**	93.7	93.9	93.7	93.7	94.2	94.3	94.3	94.3	94.3	94.4
**NY/150/2000/H3N2/NA/II**	94.5	94.7	94.5	94.5	95	95.1	95.1	95.1	95	95.1

More detailed analysis of HA and NA genes revealed that the 4 Group 2 viruses could also be grouped with Cluster IV viruses. Group 2 was most similar to the 2 Ontario turkey H3N2 variants detected in 2011 [6], with 99.6% to 99.7% and 99.4% to 99.6% identity on HA (Table 1) and NA (Table 2) genes, respectively. Interestingly, when compared to published standards, the HA gene of these 4 Group 2 viruses showed a high identity (at 98.6% to 98.8%) (Table 1) with the 2010 Quebec swine isolates. However, the NA gene of viruses showed an identity of only 95.8% to 96.0% with these Quebec swine viruses (Table 2). Group 2 was also a homogenous group of viruses with 99.9% to 100% and 99.9% to 100% identity of the HA and NA genes, respectively, although these herds were not a part of the same production system.

More detailed analysis of HA and NA genes revealed that the 2 Group 3 viruses could also be grouped with Cluster IV viruses. Group 3 viruses were most similar to the original trH3N2 [A/turkey/ON/31232/2005 and A/swine/ON/33853/2005(H3N3)] viruses detected in 2005 [3], with 96.8% to 97.1% and 98% to 98.1% identity of HA and NA genes, respectively. Interestingly, when compared to published standards, the HA and NA genes of this group of viruses showed maximum identity of 95.9% and 97.4% of the HA and NA gene, respectively, with any of the recent Canadian isolates available at the time of this study (Tables 1 and 2). This was also a homogenous group of viruses, with 99.5% and 99.9% identity of HA and NA genes, respectively, but both isolates were from a single herd.

The relatedness of each internal gene segment from each of the 10 Ontario H3N2 viruses was compared with published influenza A virus sequences. More detailed analysis of internal genes showed that the 4 Group 1 viruses indeed were the most similar to the original trH3N2 virus from 2005. The 4 Group 2 viruses were most similar to the Ontario turkey H3N2 variants with PB1 and NS genes originating from trH3N2 virus and M, PB2, PA, and NP genes originating from A(H1N1)pdm09 virus. For the 2 Group 3 viruses, all internal genes were genetically related to A(H1N1)pdm09. Phylogenetic trees for the internal gene segments are presented in Additional file 2.

### HA1 amino acid analysis

**Figure 3:**
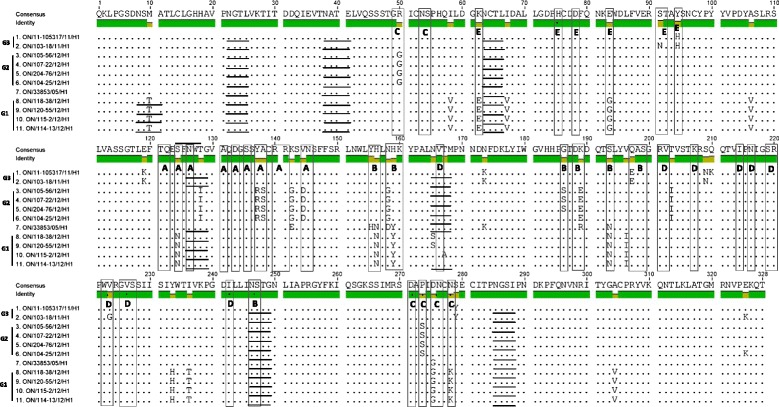
**Alignment of the 10 Ontario H3 HA1 amino acid sequences without signal peptide.** Amino acids of the HA1 subunit of the 10 Ontario H3N2 isolates and prototype cluster IV trH3N2 virus [A/swine/ON/33853/2005]. Residues shown in boxes represent previously identified antigenic sites A, B, C, D and E; respectively. Potential glycosylation sites are underlined.

In order to determine whether the aa changes occurred in any of the previously identified antigenic sites A, B, C, D and E [7], we aligned the aa sequences of the 10 Ontario H3N2 viruses and compared them with the prototype cluster IV H3N2 virus [A/swine/Ontario/33853/2005/(H3N2)]. Antigenic sites are shown in Figure 3. When compared to the known cluster IV H3N2 virus[A/swine/Ontario/33853/2005/(H3N2)], the 4 Ontario H3N2 viruses classified in Group 1 had 8 aa changes within 4 antigenic sites, A(1), B(3), C(2) and E(2). The 4 Group 2 viruses had 8 aa changes within 3 antigenic sites, A(3), B(3) and C(2), and the 2 Group 3 viruses had 4 aa changes within 4 antigenic sites, B(1), D(1) and E(2).

The Ontario H3N2 viruses from this study exhibited some unique changes at antigenic site B (Figure 3). Amino acids at position 155–160 (HNLDYK) related to prototype cluster IV virus were changed to YNLNYK (Group 1), YHLGHK (Group 2) and YHLNHK (Group 3). The receptor binding site (RBS) of the Ontario isolates examined in this study was conserved. The amino acids Y98, G134, S136, W153, H183, Y195, G225, and S227 were the same for the Ontario isolates and the prototype IV H3N2 virus (Figure 3). Identical amino acids of receptor binding pockets were also observed between isolates detected in this study and the turkey isolates reported previously and included in the analysis. The motif, N-X-T/S (X cannot be a proline) of the N-glycosylation site also has been determined (Figure 3). Representative of cluster IV, the H3N2 [A/swine/Ontario/33853/2005/(H3N2)] virus had 7 potential glycosylation sites at positions 22, 38, 63, 126, 165, 246, and 285 (underlined in Figure 3). Seven potential glycosylation sites have been predicted for Group 1 at position 8, 22, 38, 63, 126, 246, and 285. In this group one virus (A/swine/Ontario/114-13/2012(H3N2) had an additional potential glycosylation site at residue 165. Six glycosylation sites have been predicted for Group 2 (22, 38, 63, 165, 246, and 285) and seven (22, 38, 63, 126, 165, 246, and 285) for Group 3 Ontario H3N2 viruses (underlined in Figure 3).

### Resistance-associated mutations

**Figure 4:**
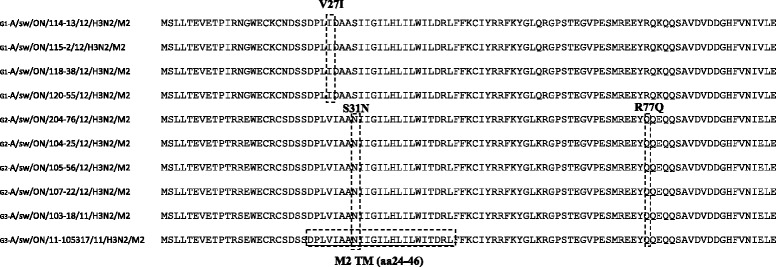
**Alignment analysis of M2 sequences of 10 Ontario swH3N2 isolates showing amino acid substitution S31N and V27I within the transmembrane domain of M2 protein, and R77Q substitution.**

Two classes of antiviral drugs are currently approved for prophylaxis and treatment of influenza A viruses in humans: adamantane derivatives (amantadine and rimantadine) and neuraminidase inhibitors (NAIs) (zanamivir and oseltamivir). To determine resistance-associated mutations to these two classes of drugs, we aligned and analyzed the aa sequences of the M and NA proteins of all 10 Ontario swH3N2 isolates (Figure 4). The most common mutation conferring resistance to adamantanes is an aa change from Ser to Asn at residue 31 (S31N) located in the transmembrane domain of the M2 protein. Sequence alignment analysis has shown that 6 of 10 Ontario swH3N2 isolates (Group 2, 3) do have the S31N mutation in the M2 protein, suggesting that Ontario swH3N2 isolates can be expected to offer resistance to amantadine and rimantadine (Figure 4). Besides the S31N mutation, additional substitutions have been traced. The R77Q substitution was observed only among 6 Ontario swH3N2 isolates with the S31N mutation. The remaining 4 isolates without S31N mutation exhibited a V27I substitution (Figure 4).

Resistance-associated mutations within the neuraminidase E119V, R292K and N294S, responsible for reduced susceptibility to oseltamivir, were not detected in any of the Ontario swH3N2 sequences (data not shown).

### Signature residues

Influenza virus segment 2 encodes 3 proteins, PB1, PB1-F2 encoded by an alternative open reading frame (ORF) of segment 2, and PB1 N40 N-terminally truncated version of the PB1 protein. Phylogenetic analysis of 32 PB1 aa sequences showed that PB1-F2 of all 10 Ontario H3N2 viruses belongs to lineage D (Additional file 3).

**Figure 5:**
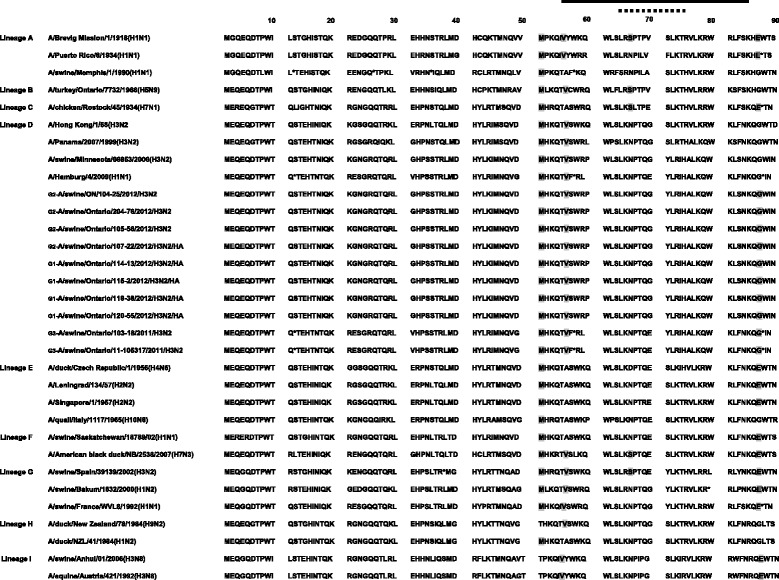
**Presentation of PB1-F2 variants of mammalian and avian influenza A viruses.** Nine PB1 lineages from A to I have been described and some selected sequences within these lineages are presented. Lines at the top of the Figure represent amino acids of the predicted helical region (black) and the putative mitochondrial targeting sequence (dashed). Amino acids that are considered to enhance viral pathogenesis are marked in grey. The first stop codon has been shown by asterisk and following stop codons are indicated by subsequent asterisk.

Eight of 10 Ontario H3N2 viruses (Figure 5) had complete 90 aa PB1-F2 fragments (long bar). The remaining 2 viruses (A/sw/Ontario/103-18/2011(H3N2)) and (A/sw/Ontario/11-105317/2011(H3N2)) from Group 3 expressed a truncated form of PB1-F2 containing 11 amino acids (short bar), lacking the mitochondrial targeting sequence (MTS) located in the C-terminal region. Further sequence analysis of Ontario H3N2 PB1-F2 proteins revealed the absence of the N66S mutation which was previously reported in the H1N1, 1918 PB1-F2 and pathogenic avian H5N1 viruses and found to be associated with a gain in virulence. In addition to N66S, further amino acids T51, V56 and E87 might also enhance pathogenicity. As can be seen from Figure 5 the aa sequences of Ontario H3N2 PB1-F2 all contain V56, but substitutions T51M and E87G, previously associated with lower pathogenicity, have also been determined [8].

Further, we assessed PB2 signature amino acids, which greatly influences host range. The PB2 key signature aa at position 627 is an important determinant of virulence and host specificity [9-11]. This analysis demonstrated the absence of a PB2 E627K mutation, but revealed 590-91SR and 271A mutations in all Ontario H3N2 viruses (data not shown). The H1N1 2009 pandemic viruses also had PB2 627E and carried PB2 590-91SR and 271A mutations. Such a distribution of mutations might compensate for PB2 627E and adapt the viruses for efficient replication in mammals.

Signature residues of the PA protein have also been assessed and showed the absence of PA 100A that is present in human influenza A viruses that caused the pandemic of 1977 A(H1N1), 1968 A(H3N2), and 1957 A(H2N2). Instead of 100A, Ontario swH3N2 isolates had PA 100 V. Additional mutations, PA 356R and 409 N associated with past influenza pandemics, including 2009 (H1N1), 1977 (H1N1), 1968 (H3N2) and 1997 (H5N1), respectively, have also been detected in Ontario swH3N2 isolates (data not shown).

Close attention has been paid to the NP protein as a part of the helical genomic ribonucleoprotein complex with an important role in viral RNA replication and cross-species transmission. The NP protein with isoleucine at position NP 100, associated with increased human-to-human transmissibility, found in both 2009 and 1918 H1N1 pandemic viruses, has not been detected in Ontario swH3N2 isolates. Instead of an isoleucine residue, glycine was detected (data not shown).

## Discussion

In this report we describe the genetic and predicted antigenic characterization of 10 Ontario H3N2 viruses isolated from swine in 2011 and 2012. On the basis of these results it can be concluded that at least three different groups of H3N2 viruses were circulating in Ontario swine during that time.

Group 1 represents viruses similar to the original trH3N2 viruses with gene segments of avian (PB2, PA), human (PB1, HA, NA) and classical swine (NP, M, NS) influenza virus origin. These 4 trH3N2 genotype viruses, with a mix of classical avian, human and swine virus lineages, emerged in 1998 and rapidly spread throughout the US swine population [12,13]. In 2005, the spread of these viruses to Canada were reported in Canadian swine, turkey, and mink populations [3,4]. Group 2 represents viruses with HA, NA, PB1, and NS genes closely related to trH3N2 viruses. The remaining internal genes are genetically related to A(H1N1)pdm09 virus. These 4 Ontario H3N2 viruses exhibited the same gene assemblage as those found in trH3N2 influenza viruses isolated from breeder turkey flocks in Southern Ontario during 2011 (FAV-009 and FAV-010). Group 3 represents viruses with HA and NA genes closely related to trH3N2 viruses, with all internal genes genetically related to A(H1N1)pdm09. Although structurally the same gene assemblage has been observed as in the recent Quebec isolates [14], more detailed analysis showed that some gene segments in this group of 2 viruses were most similar to the Ontario H3N2 historical isolates from 2005, suggesting that these viruses likely were of Ontario origin, and not introduced from Quebec, despite frequent swine movement between the two Canadian provinces. In 2009, a previously unrecognized H1N1 swine-origin subtype comprising a gene combination of North American and Eurasian IAV-S was isolated from humans in the US and Mexico [15]. This virus spread globally as the first pandemic of the 21th century. Shortly after the virus was first isolated from humans in the USA and Mexico, human-to-animal transmission was reported in Canada by Howden et al. [16] and Berhane et al. [17]. Virus isolates from Canadian pigs were phylogenetically similar to A(H1N1)pdm09 virus [16,18], which is often detected in the Canadian swine population [19]. The characterization of the Ontario H3N2 viruses clearly indicates reassortment of gene segments between the North American swine trH3N2 from Cluster IV and the A(H1N1)pdm09. Structurally similar viruses have been observed on multiple occasions since A(H1N1)pdm09 emerged in 2009 [4,20]. More recently, Kitikon et al. [21] reported at least ten unique groups of H3N2 reassortants based on H3N2/H1N1pdm(09) gene combination. Findings in this study have implications for animal and public health, because H3N2 viruses with the matrix gene from H1N1pdm09 were implicated in influenza outbreaks in people attending agricultural fairs in US in 2012 [22-24], showed efficient droplet transmission in experimental studies on ferrets [25], and increased person-to-person transmissibility in modelling studies based on outbreak data in people [26].

The percentage of aa identity of HA1 between the prototype Cluster IV trH3N2 virus [A/swine/Ontario/33853/2005/(H3N2)] and 10 Ontario H3N2 viruses ranged from 94.5% to 95.7%. We detected 8 aa changes at 4 antigenic sites (A, B, C and E) for Group 1 viruses, 8 aa changes at 3 antigenic sites (A, B and C) for Group 2, and 4 aa changes at 3 antigenic sites (B, D and E) for Group 3. According to Wilson and Cox [27], a drift variant with ≥4 aa changes at ≥2 of 5 antigenic sites could be of epidemiologic importance. Additionally, Wood et al. [28] and Kodihalli et al. [29] have shown that 1 to 3 aa changes in the HA1 of H1N1 and H3N2 viruses can reduce cross reactivity and efficacy of inactivated vaccine. This leaves open the possibility that several variants of H3N2 could co-circulate in the same population. However, more detailed serological studies and field studies should be done to better appreciate the potential for such co-circulation or the frequency with which this occurs.

Although, the Ontario H3N2 viruses from this study shared 5 potential N-linked glycosylation sites at positions 22, 38, 63, 126, and 285 with the closely related cluster IV A/swine/Ontario/33853/2005/(H3N2) virus, Group 2 (4 viruses) lacked a potential glycosylation site at position 126, Group 1 (4 viruses) had an additional potential glycosylation site at position 8, and 3 viruses in Group 1 lacked a glycosylation site at position 165. The existence of N-glycosylation is essential for viral membrane glycoproteins. The N-glycans encode required information for folding, maturation, transport or degradation of proteins [30]. Glycosylation seems to be one mechanism by which a virus masks its epitopes and escapes neutralization by the host antibodies [31,32]. However, the study of Vigerust et al. [33] linked increased glycosylation of influenza viruses with decreased virulence.

The M2 is the target of the anti-influenza drugs amantadine and rimantadine. Resistance-associated mutation to adamantanes S31N has been detected in 6 Ontario H3N2 viruses indicating resistance to amantadine and rimantadine. According to our findings at least 6 Ontario swH3N2 viruses out of 10 evaluated can be expected to offer resistance to amantadine and rimantadine.

In conclusion, this study showed that at least 3 different variants of H3N2 viruses circulated in Ontario swine in 2011 and 2012. Group 1 viruses are most closely related to trH3N2 viruses from 2005 [3]. Group 2 viruses are closely related to H3N2 viruses that were previously reported in Ontario turkeys [6]. It is important to note that there were no clear epidemiological linkages among swine and turkey farms with detected viruses, although no detailed disease investigations were performed as a part of this study. Group 3 viruses are most closely related to swH3N2/A(H1N1)pdm09 reassortant viruses from swine in the province of Quebec, but are likely of Ontario origin. According to amino acid sequence analysis of the M2 protein, Ontario H3N2 viruses can be expected to offer resistance to adamantane derivatives, but not to neuraminidase inhibitors.

## Materials and methods

### Study populations

Invitation for participation in the study was distributed through the Ontario Association of Swine Veterinarians’ email distribution list. Inclusion criterion for these herds was the current or recent presence of clinical signs suggestive of infection with influenza A virus as indicated by a herd veterinarian. Twenty-one Ontario swine herds were sampled between November 2011 and May 2013, with the majority of 18 herds sampled in 2012. Groups and animals showing clinical signs suggestive of influenza were targeted for sampling. Dacron-tipped nasal swabs were collected from both nostrils of individual animals, deposited in PBS, and transported to a virology laboratory (Department of Pathobiology, University of Guelph). Nasal swabs were either tested within 24 h or frozen at −80°C before being inoculated in MDCK cells. The originally planned sample size was 100 per herd, sufficient to detect a prevalence of infection between 2% and 3%. However, actual sample size varied depending on the prevalence of clinical signs and resources available for sampling.

In twelve of those 21 herds an additional 30 nasal swabs were collected from individual animals with clinical signs indicative of influenza. These samples were transported in 2 ml of VTM and submitted to the Animal Health Laboratory (AHL, University of Guelph, ON, Canada). The samples were then pooled 3:1 and tested with a rtRT-PCR directed to the matrix protein of influenza A viruses.

Eleven viruses from 10 herds that tested positive for H3N2 influenza virus were included in the full genome sequencing study. The viruses were selected purposively so that only one farm per production system was included. For 9 herds, one randomly selected virus was included; and for one herd, 2 randomly selected viruses were included.

### Samples submitted to the virology laboratory of the department of pathobiology

Swab samples were added to MDCK cells as described elsewhere [34]. Virus isolates were identified by a hemagglutination (HA) assay, as described elsewhere [34].

Viral RNA was extracted using the RNeasy Mini Kit according to the manufacturer’s instructions (Qiagen Inc., Valencia, CA, USA). Briefly, the cDNA was synthesized with Random Primer (Invitrogen, Canada Inc., Burlington, ON, Canada) and by SuperScript™ II Reverse Transcriptase, Invitrogen, Canada Inc Burlington, ON, Canada) according to the manufacturer’s instruction. The subtype was determined by reverse transcription (RT-PCR) and further characterized by full genome sequencing using 454 sequencing technology. The RT-PCR subtyping was performed on the harvested virus infected cell culture supernatant, as identified by CPE and HA. Primer pairs utilized in study for subtype determination were: HA gene (subtype H1Forw 5′-GTTCTGCTATATACATTTGC-3′, H1Rev 5′-GACCCATTAGAGCACATC-3′ or H3Forw 5′-GGA-AGT-GAC-AAC-AGC-AT-3′, H3Rev (5′-AGC-TGA-ACA-CCT-TTG-ATC-T-3′) 5′- and NA gene (subtype N1Forw 5′- TAACATCAGCAACATCAACT-3′, N1Rev GTCGCCCTCTGATTAGTT-3′ or N2 Forw 5′- GGCTCTGTTTCTCTCATC-3′, N Rev 5′- CAAGTCCTGAGCATACATA-3′). The PCR was performed with 35 cycles of 94°C for 15 s, 54°C for 30s, and 72°C for 60 s. The results of the RT-PCR assay designated the influenza virus type A virus to be either subtype H1N1 or H3N2.

### Samples submitted to the AHL

Viral RNA was extracted on an automated platform using the MagMAX™ Express-96 instrument and MagMAX™ 96 Viral RNA Isolation kit (cat#AM1836), according to the manufacturer’s instructions. To identify swine influenza A viruses a rtRT-PCR was used with matrix primers redesigned by Dr J. Pasick (CFIA, Winnipeg, MB,CA), enhanced for the detection of A(H1N1)pdm09, using SIV Forward Primer - 5′d AGA TGA GTC YTC TAA CCG AGG TCG 3′; SIV Reverse Primer - 5′d TGC AAA RAC AYY TTC MAG TCT CTG 3′. An AIV M + 64 Probe -5′FAM-TCA GGC CCC CTC AAA GCC GA-BHQ-1 3′designed by Spackman et al. [35] was used. The probe was purchased from Biosearch Technologies as Reference # SS113269-29. These were used in combination with the Ambion AgPath-ID one-step RT-PCR Kit Cat #AM1005, according to the manufacturer’s instructions.

If the matrix rtRT-PCR was positive, then the specimen was processed for virus isolation using MDCK cells and rtRT-PCR subtyping was performed on the harvested virus infected cell culture supernatant, as identified by CPE and HA using chicken, turkey or guinea pig red blood cells. Where large concentrations of virus were present in clinical samples, as evidenced by low CT values in the matrix rtRT-PCR, subtyping was attempted directly from clinical samples. However in the majority of cases clinical samples did not have high enough levels of viral RNA for successful subtyping and cell culture supernatant was used.

The first step for subtyping was initial production of complementary DNA (cDNA) from RNA extracted from MDCK or allantoic fluid culture isolates using random hexamer priming and MuLV reverse transcriptase. During the second step the cDNA was amplified using single primer pairs to type the isolate as H1N1 or H3N2. Primer pairs were complementary to the HA gene (subtype H1 -612 F5′-GGT GTG ACG GCA GCA TGC CCT-3′;H1 612 R 5′-AGC AAT GGC TCC AAA CAG ACC-3′ or H3 722 F 5′-CARATTGARGTGACHAATGC-3′; H3 722 R 5′-GGTGCATCTGAYCTCATTA-3′) and the NA gene (subtype N1 533 F 5′-CTT CCT ATC CAA ACA CCA TT-3′; N1 533 R5′-TTG CTT GGT CAG CAA GTG-3′ or N2 385 F 5′-ACGACAGATCCAGCAGTAGC-3′; N2 385R 5′-GAGCCTGTTCCATATGTACC-3′). The results of the rtRT-PCR assay designated the influenza virus type A virus to be either subtype H1N1, H3N2 or an unknown virus.

### Genomic sequencing and sequence assembly

The complete nucleotide sequences of 10 viruses were determined using the 454 GS Junior Titanium platform (Roche Applied Science, Indianapolis, IN, USA). In brief, RNA of all viruses was fragmented by ZnCl_2_ into fragments between 500 bp and 1500 bp. Sheared RNA was used for first and second strand cDNA synthesis and fragment end repair. The 454 rapid library multiplex identifier (RL MID) was ligated to the fragments according to the GS Junior Titanium cDNA rapid library preparation method (Roche) by incubating with ligase at 25C° for 10 min. Quality assessment of the RNA samples and of the DNA library was performed using the FlashGel system. The nucleotide sequence reads obtained were assembled using the Newbler (version 2.5p1) *de novo* sequence assembly software (Roche). Comparison of the *de novo* contigs by BLAST with known influenza A virus sequences allowed identification of all genes. Sanger sequencing applying targeted oligo primers was utilized to walk across gaps. BLASTN and BLASTX analyses were performed to compare the established sequences to known influenza A viruses in the NCBI database.

### Sequence analysis

To determine gene relatedness of each gene segment of the Ontario swine H3N2 isolates, we used BLAST from the GenBank database. Geneious Pro 5.5.6. has been used to determine nucleotide and amino acid (aa) sequence identities. The CLUSTAL W alignment method was applied, and an unrooted phylogenetic tree of the HA gene was constructed by using the distance-based neighbor-joining method. To predict N-linked glycosylation sites (Asn-X-Ser/Thr, where X is any aa except Pro) we applied the NetNGlyc 1.0 server.

The University of Guelph Animal Care Committee and the Ethics Committee approved this study.

## Additional files

Additional file 1
**Genome constellations identified in Ontario H3N2 viruses isolated from swine in 2011 and 2012.** Green = trH3N2. Red = pandemic H1N1. Additional file 2
**Phylogenetic trees for the NA gene and the six internal gene segments (A) PB2; (B) PB1; (C) PA; (D) NP; (E) NA; (F) M; (G) NS of 10 Ontario H3N2 viruses.**
Additional file 3
**Phylogenetic analysis of 32 PB1 sequences.** Representatives of the nine PB1 lineages ranging from A to I have been selected. The pandemic strains of 1918 (H1N1), 1957 (H2N2), 1968 (H3N2) and 2009 (H1N1) are marked with asterisk.
