# Supplementary material for: Molecular characterization of H3N2 influenza A viruses isolated from Ontario swine in 2011 and 2012
Source: Virol J. 2014 Nov 22;11:194. doi: 10.1186/s12985-014-0194-z (PMC4245826; doi:10.1186/s12985-014-0194-z)
Supplement: Additional file 1 — Genome constellations identified in Ontario H3N2 viruses isolated from swine in 2011 and 2012. Green = trH3N2. Red = pandemic H1N1. [file 12985_2014_194_MOESM1_ESM.doc]

| **Ontario H3N2 isolates** | **HA** | **NA** | **PB1** | **PB2** | **NP** | **PA** | **NS** | **M** |
| --- | --- | --- | --- | --- | --- | --- | --- | --- |
| **ON/103-18/11/H3N2** |  |  |  |  |  |  |  |  |
| **ON/11-105317/11/H3N2** |  |  |  |  |  |  |  |  |
| **ON/104-25/12/H3N2** |  |  |  |  |  |  |  |  |
| **ON/204-76/12/H3N2** |  |  |  |  |  |  |  |  |
| **ON/105-56/12/H3N2** |  |  |  |  |  |  |  |  |
| **ON/107-22/12/H3N2** |  |  |  |  |  |  |  |  |
| **ON/114-13/12/H3N2** |  |  |  |  |  |  |  |  |
| **ON/115-2/12/H3N2** |  |  |  |  |  |  |  |  |
| **ON/118-38/12/H3N2** |  |  |  |  |  |  |  |  |
| **ON/120-55/12/H3N2** |  |  |  |  |  |  |  |  |

**G3**

**G2**

**G1**

**Additional file 1** Genome constellations identified in Ontario H3N2 viruses isolated from swine in 2011 and 2012.

Green=trH3N2

Red=pandemic H1N1
