# Supplementary material for: Molecular characterization of H3N2 influenza A viruses isolated from Ontario swine in 2011 and 2012
Source: Virol J. 2014 Nov 22;11:194. doi: 10.1186/s12985-014-0194-z (PMC4245826; doi:10.1186/s12985-014-0194-z)
Supplement: Additional file 2 — Phylogenetic trees for the NA gene and the six internal gene segments (A) PB2; (B) PB1; (C) PA; (D) NP; (E) NA; (F) M; (G) NS of 10 Ontario H3N2 viruses. [file 12985_2014_194_MOESM2_ESM.doc]

**A) Segment 1, PB2**


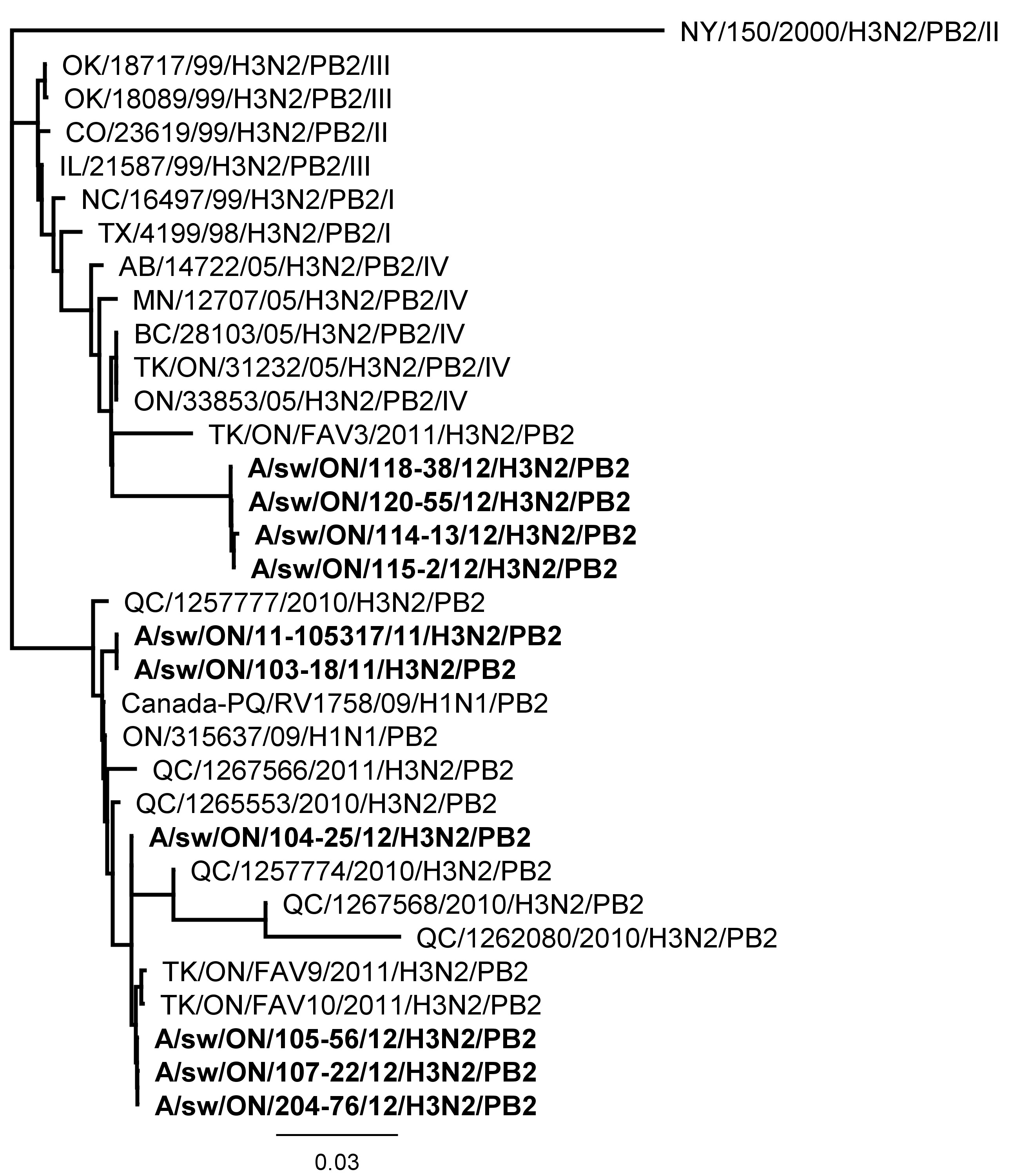


**B) Segment 2, PB1**


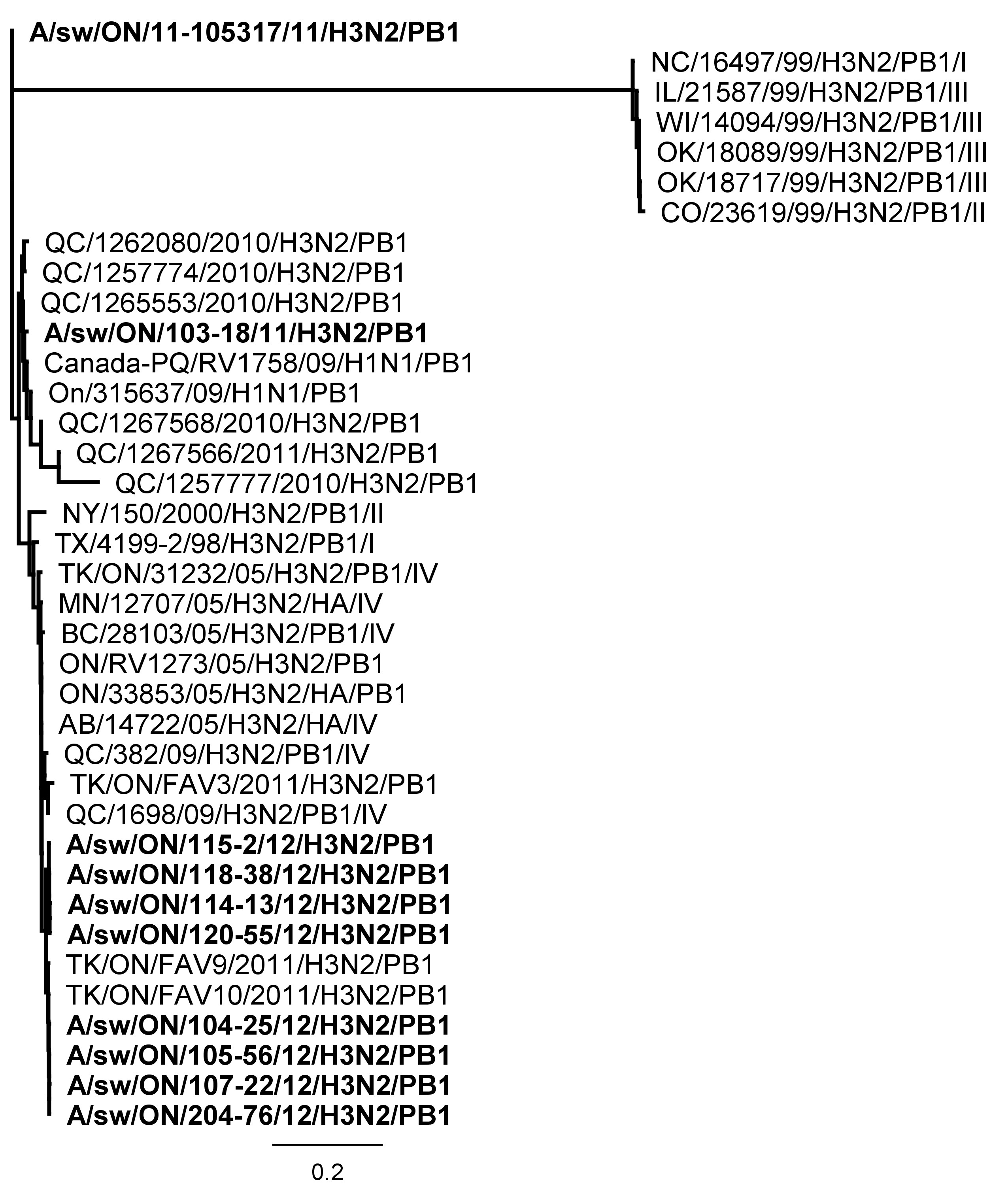


**C) Segment 3, PA**


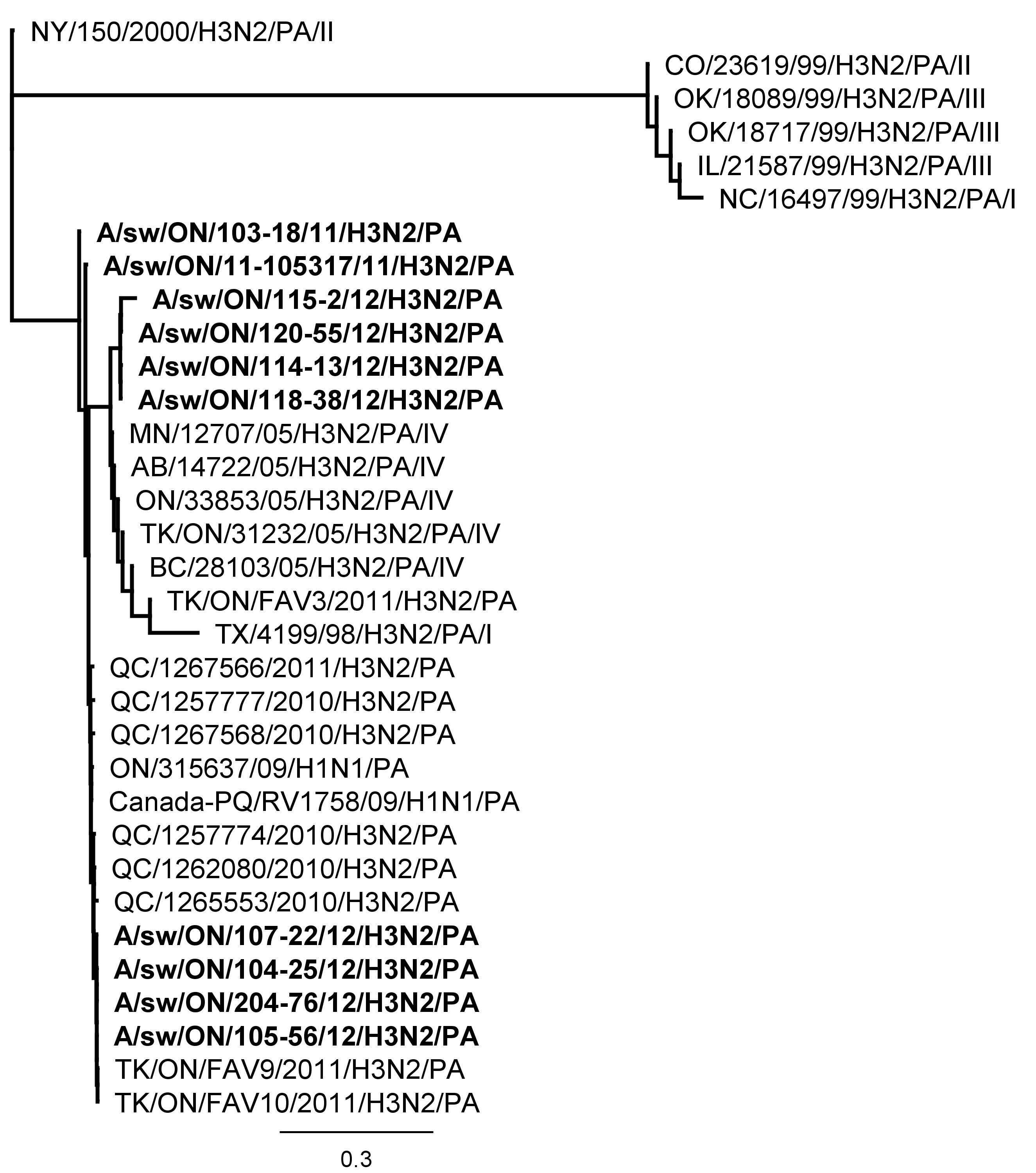


**D) Segment 5, NP**


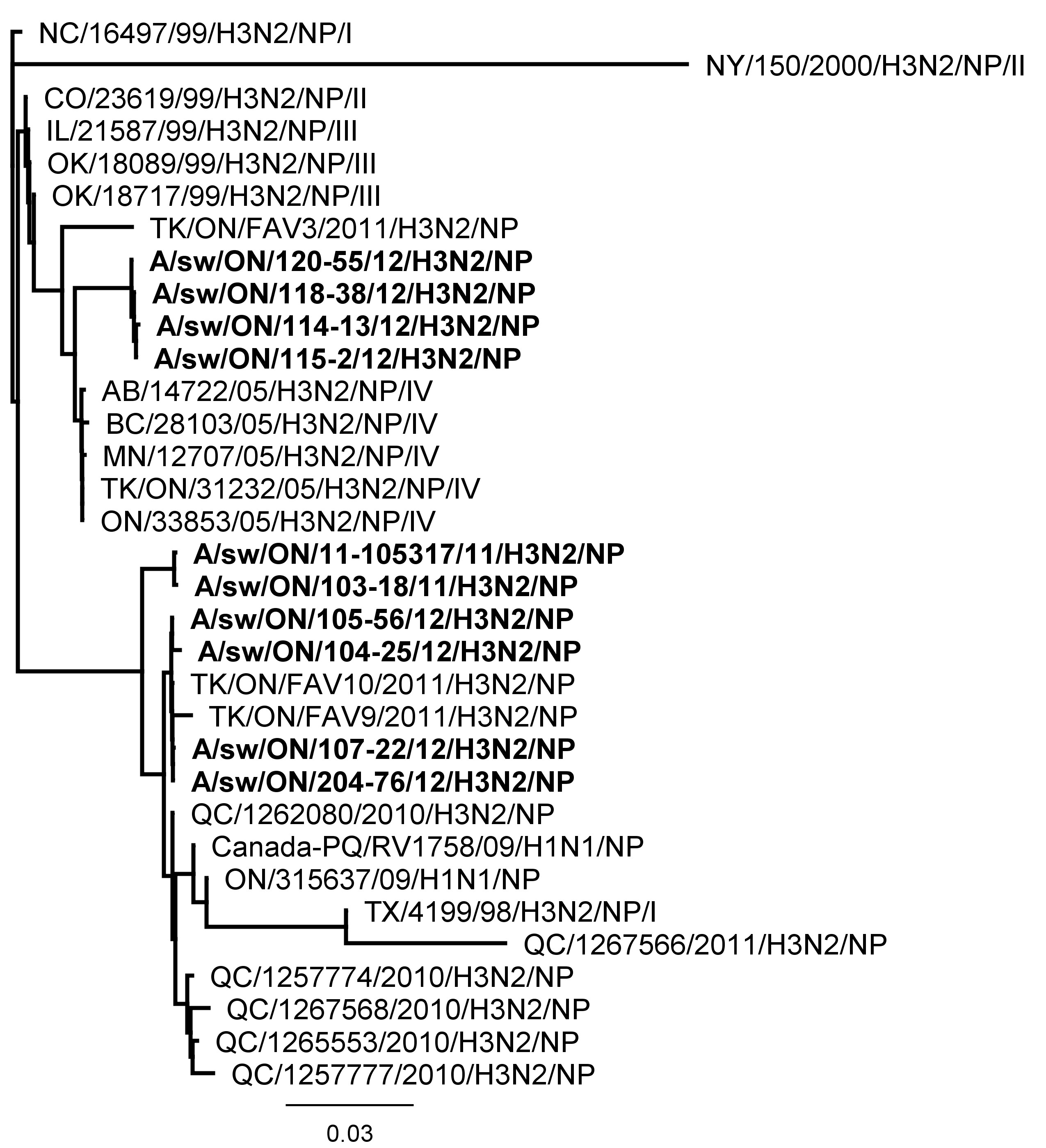


**E) Segment 6, NA**


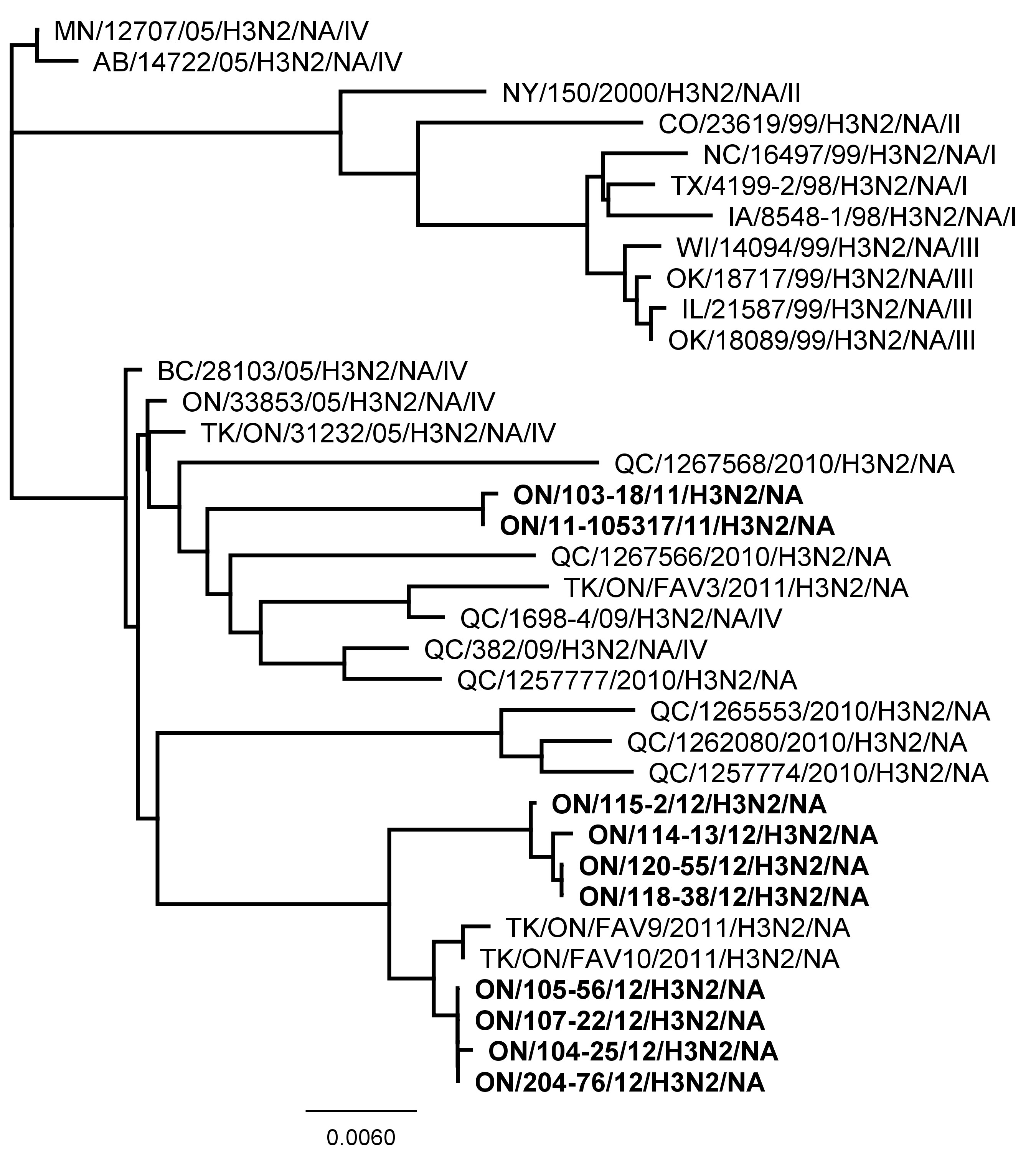


**F) Segment 7, M**


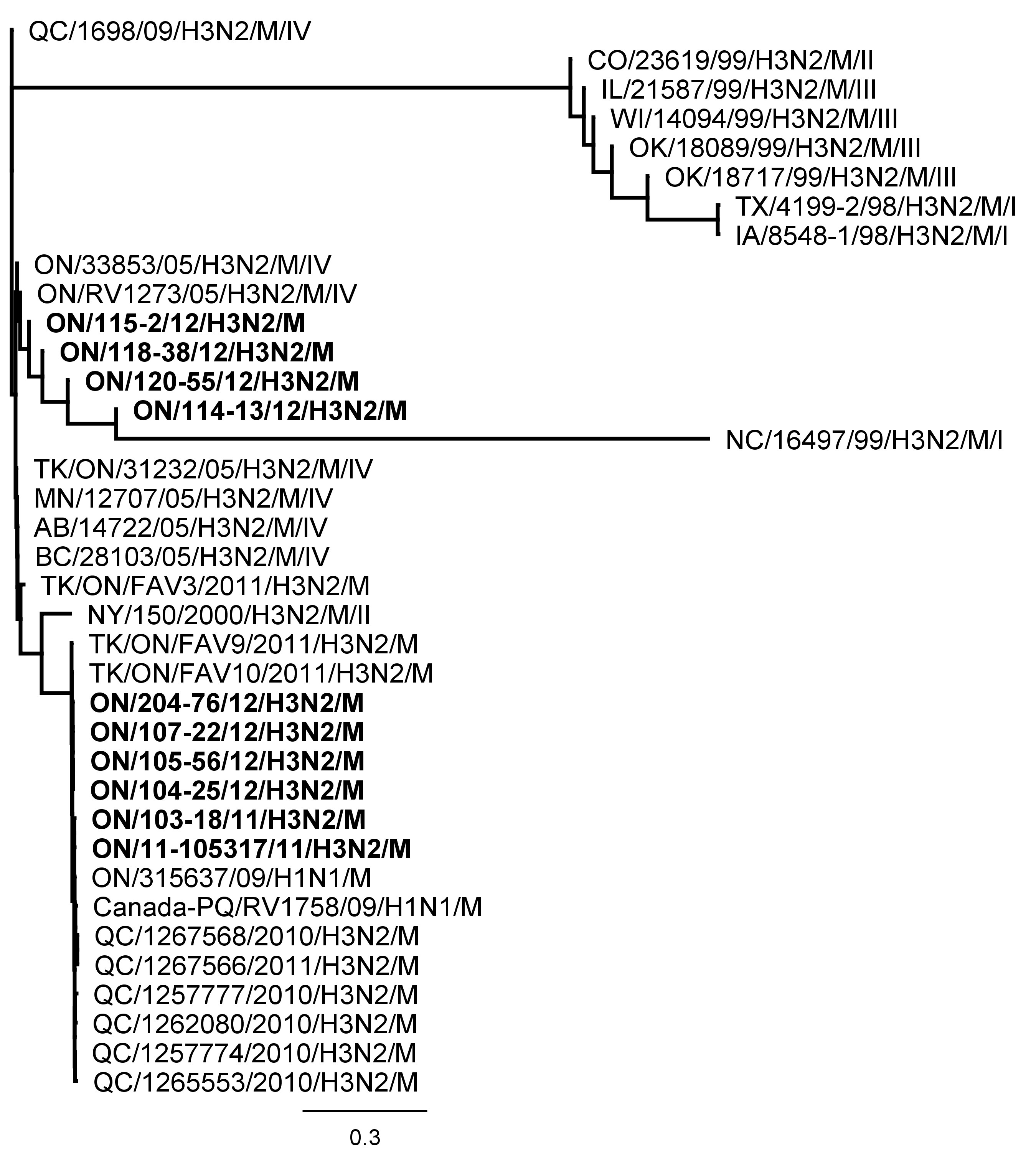


**G) Segment 8, NS**


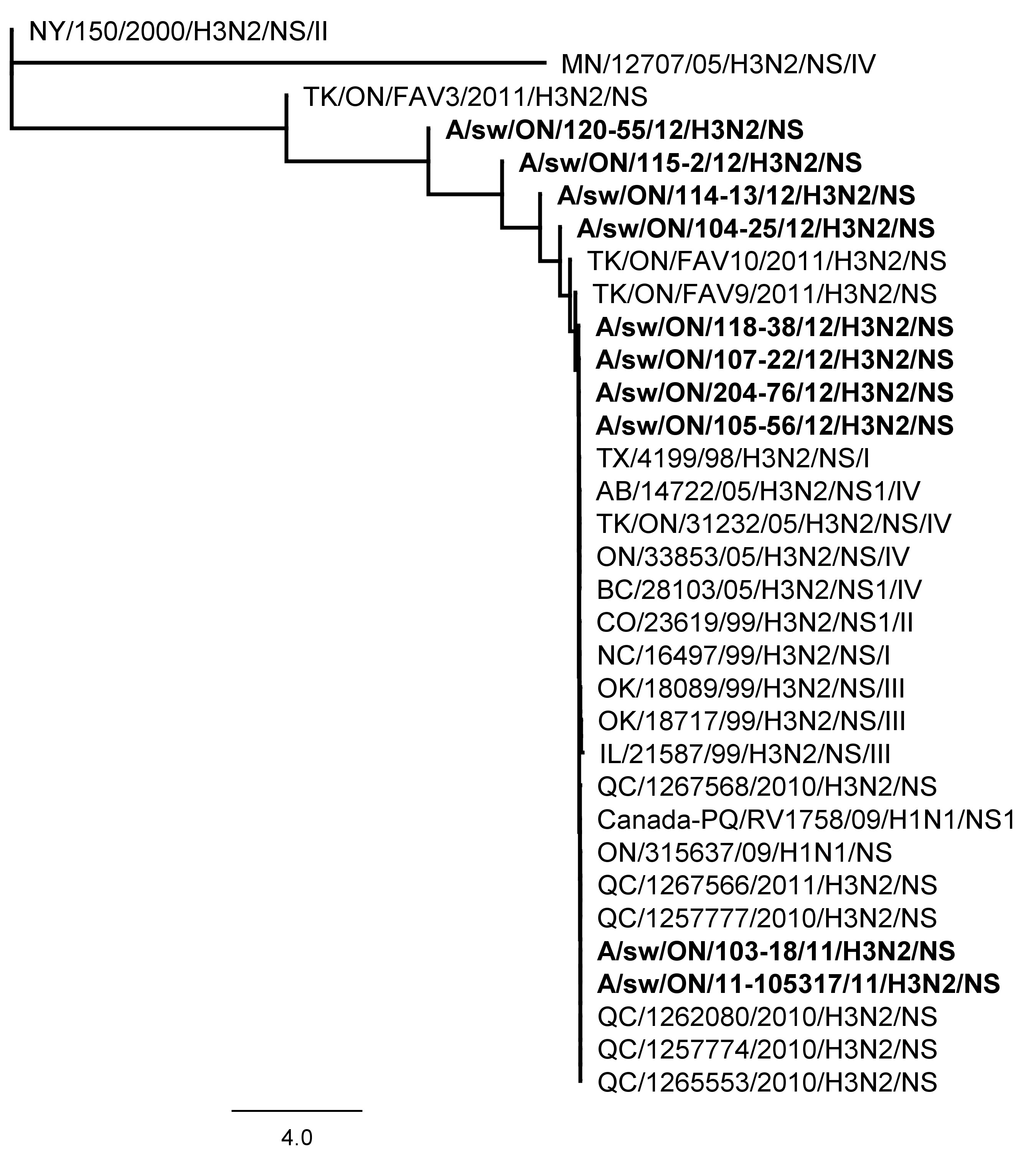


**Additional file 2** Phylogenetic trees for the NA gene and the six internal gene segments (A) PB2; (B) PB1; (C) PA; (D) NP; (E) NA; (F) M; (G) NS of 10 Ontario H3N2 viruses.
