# Supplementary material for: Molecular characterization of H3N2 influenza A viruses isolated from Ontario swine in 2011 and 2012
Source: Virol J. 2014 Nov 22;11:194. doi: 10.1186/s12985-014-0194-z (PMC4245826; doi:10.1186/s12985-014-0194-z)
Supplement: Additional file 3 — Phylogenetic analysis of 32 PB1 sequences. Representatives of the nine PB1 lineages ranging from A to I have been selected. The pandemic strains of 1918 (H1N1), 1957 (H2N2), 1968 (H3N2) and 2009 (H1N1) are marked with asterisk. [file 12985_2014_194_MOESM3_ESM.doc]

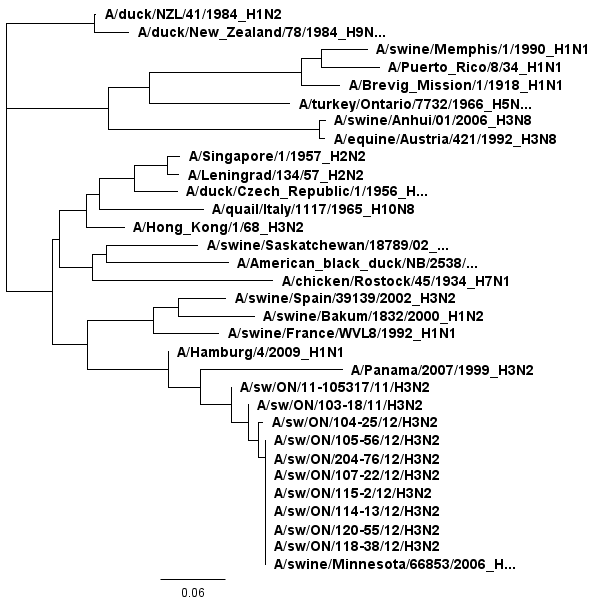


**H**

**F**

**G**

**E**

**A**

**B**

**I**

**C**

**D**

*****

*****

*****

*****

**Additional file 3** Phylogenetic analysis of 32 PB1 sequences. Representatives of the nine PB1 lineages ranging from A to I have been selected (Krumbholz et al., 2011). The pandemic strains of 1918 (H1N1), 1957 (H2N2), 1968 (H3N2) and 2009 (H1N1) are marked with asterisk
